# Where is the Guidewire? Confirmation of Central Catheter Placement in the Brachiocephalic Vein Using Y-shape Visualization by Ultrasound

**DOI:** 10.7759/cureus.4124

**Published:** 2019-02-23

**Authors:** Tayfun Aydın, Onur Balaban, Rabia Koçulu, Murat Emre Tokur

**Affiliations:** 1 Anesthesiology, Kutahya Health Sciences University, Kutahya, TUR; 2 Internal Medicine, Kutahya Health Sciences University, Kutahya, TUR

**Keywords:** central venous catheter, endocavity micro-convex probe, ultrasound, y-shape visualization

## Abstract

Central venous catheter placement with ultrasound guidance improves the success rate and reduces the number of puncture attempts and complications. Y-shape visualization of central veins using an endocavity micro-convex ultrasound probe is a new technique, which has been used for brachiocephalic vein cannulation. Since the jugular, subclavian, and brachiocephalic veins can be visualized in a single view using the Y-shape technique, it can also be used to confirm the correct placement of the catheter or guidewire. We aimed to present a case in which the location of the guidewire was verified by Y-shape visualization with an endocavity micro-convex probe after a cannulation attempt. Successful internal jugular vein catheterization was achieved with the assistance of the Y-shape imaging technique and the patient was avoided from multiple cannulation attempts.

## Introduction

Central venous catheterization (CVC) is an invasive procedure performed perioperatively in emergency departments and critical care units due to various indications [1]. However, it can cause serious mechanical complications with an incidence of up to 12% [2]. Among these complications, carotid placement of the catheter may lead to serious problems, including excessive bleeding or local hematoma. Ultrasound (US)-guided CVC has become the standard for central venous access [3]. In the US-guided CVC technique, linear probes have been accepted as the classic approach and widely used, with the advantage of high resolution.

The use of an endocavity micro-convex probe for internal jugular vein catheterization was first proposed by Phelan et al. [4]. Endocavity micro-convex probes provide sufficient resolution for both superficial tissues, such as the internal jugular vein (IJV), and deep tissues, such as the brachiocephalic vein, with a higher tissue penetration. With this technique, the internal jugular vein, subclavian vein (SV), and brachiocephalic vein (BV) can be visualized together as a Y-shape [4-5]. The guidewire can be advanced within the brachiocephalic vein by real-time visualization and the catheter position in the BV can be visualized as well [6-7].

We present a case of internal jugular vein cannulation where a guidewire was placed using a short-axis US-guided technique and the location of the guidewire in the brachiocephalic vein was verified by Y-shape visualization, which was not possible to visualize with a linear probe. Our aim is to emphasize the usefulness of Y-shape visualization using an endocavity micro-convex probe for the confirmation of guidewire placement in the brachiocephalic vein.

## Case presentation

**Figure 1:**
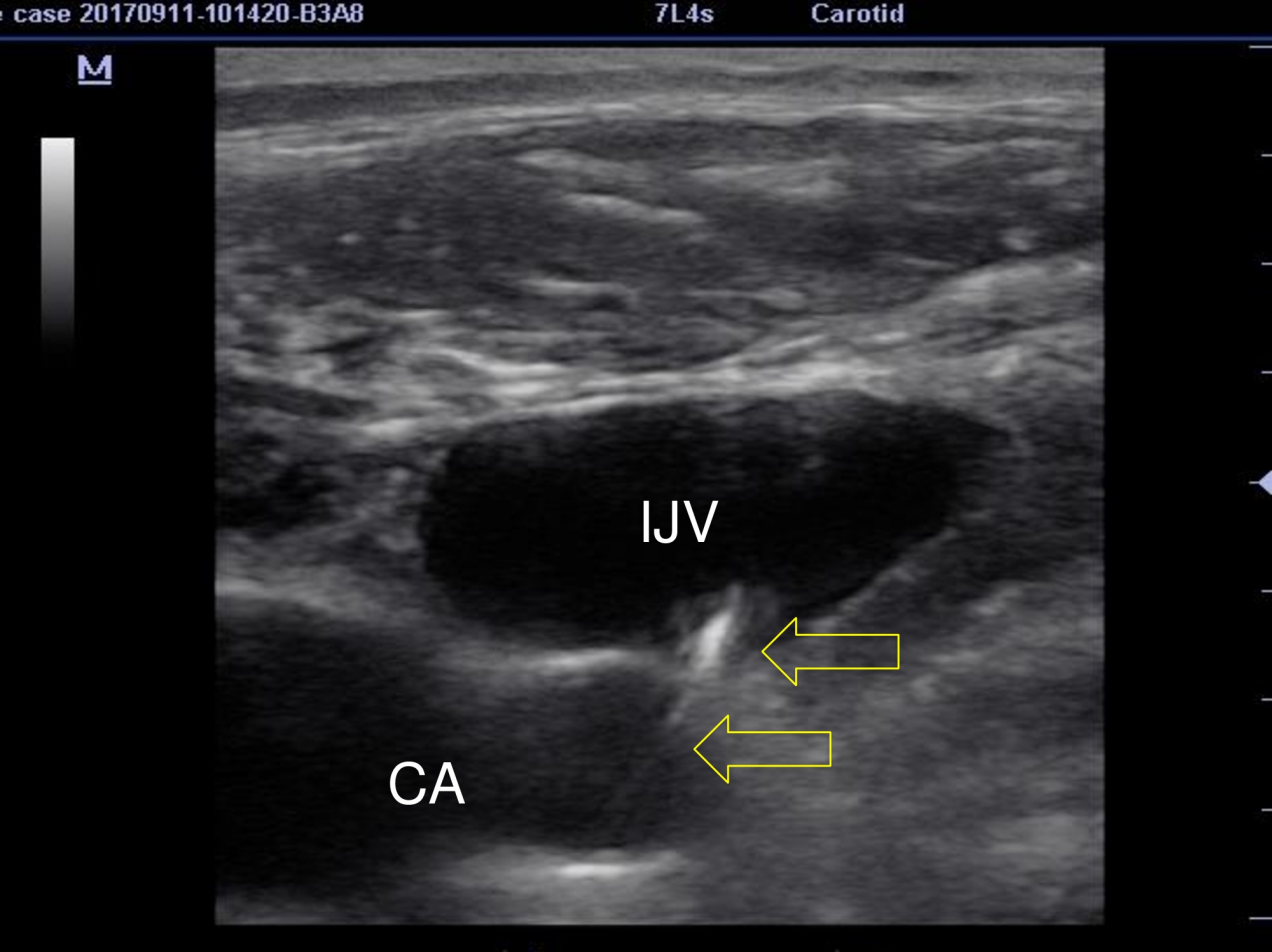
Image of the carotid artery and internal jugular vein obtained by the linear probe The guidewire (arrows) seems to puncture and is located in the carotid artery ( IJV: internal jugular vein, CA: carotid artery).

A 58-year-old obese female patient with hypertension, diabetes mellitus, and a 32.7 kg/m^2^ body mass index (BMI) was admitted to the operating room. The patient was planned to undergo gastric bypass surgery. After standard monitoring (electrocardiogram and pulse oximetry) and arterial line placement, the induction of general anesthesia was achieved using 2 mg/kg propofol, 2 µg/kg fentanyl, and 0.6 mg/kg rocuronium intravenously. In our clinic, central venous catheters are routinely placed to the IJV for patients undergoing bariatric surgery. Thus, we planned to place a central venous catheter in the right IJV under ultrasound guidance with a short axis (out-of-plane) technique using a linear US probe. In one attempt, we accessed the jugular vein and placed the guidewire, however, the aspirated blood color appeared bright red, which made us suspicious of a carotid artery cannulation. We checked with the linear probe to verify the location of the guidewire in the IJV. With a linear probe view, there was an image showing that the guidewire was placed in the carotid artery (Figure 1).

**Figure 2:**
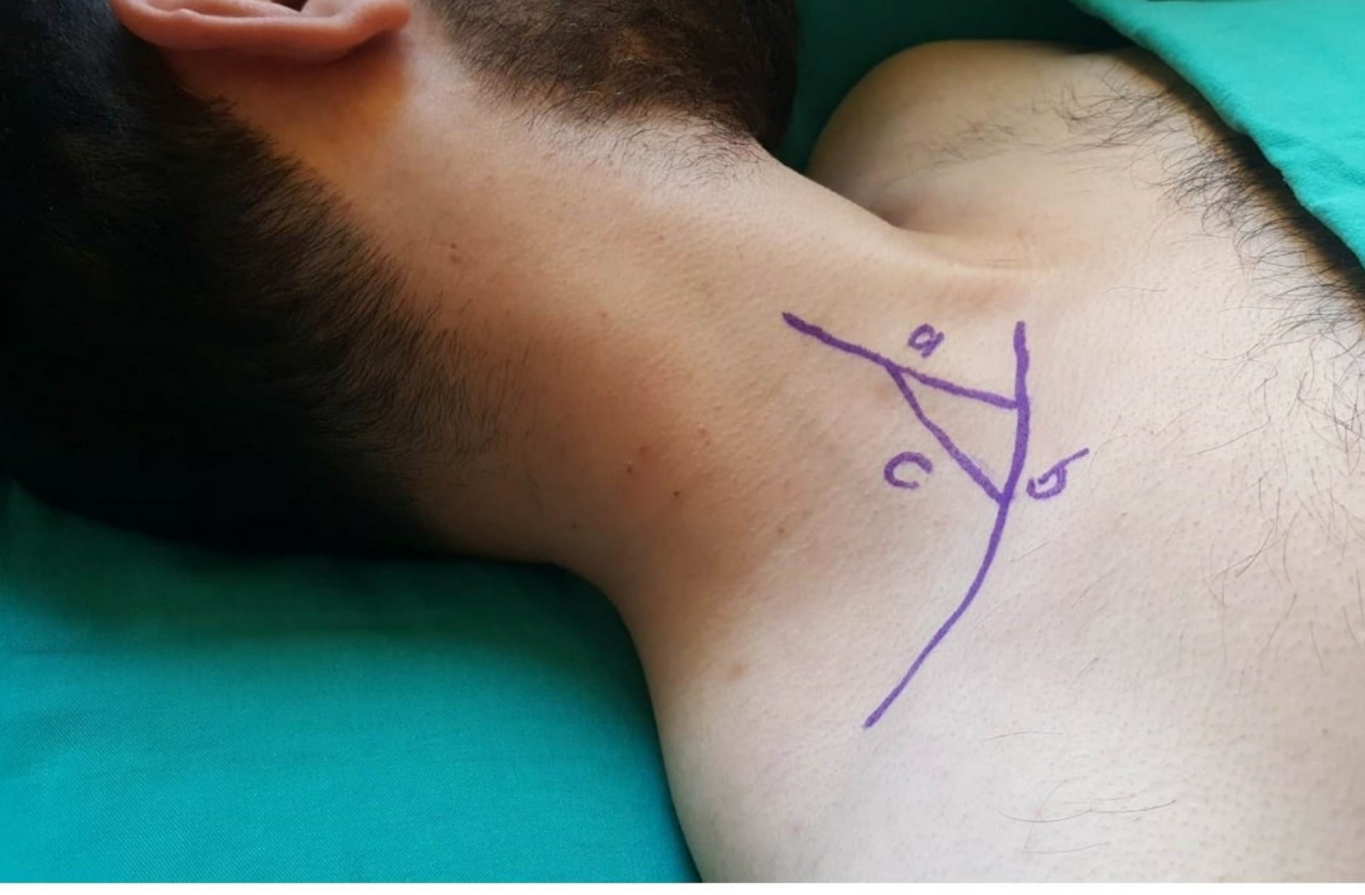
Photograph of the omoclavicular acoustic window in a volunteer a: clavicular head of sternocleidomastoid muscle, b: superior border of clavicula, c: inferior belly of omohyoid muscle

This image could be just a reverberation artifact of the guidewire extending into the artery and not actually the wire itself. We could not verify the placement of the guidewire in the jugular vein with manipulation under US visualization. For this reason, we thought that the guidewire should also be checked with an endocavity micro-convex probe before the dilatation of the vessel. We visualized the right brachiocephalic vein using an endocavity micro-convex probe by placing it in the triangular area, which is called the omoclavicular acoustic window (Figure 2).

**Figure 3:**
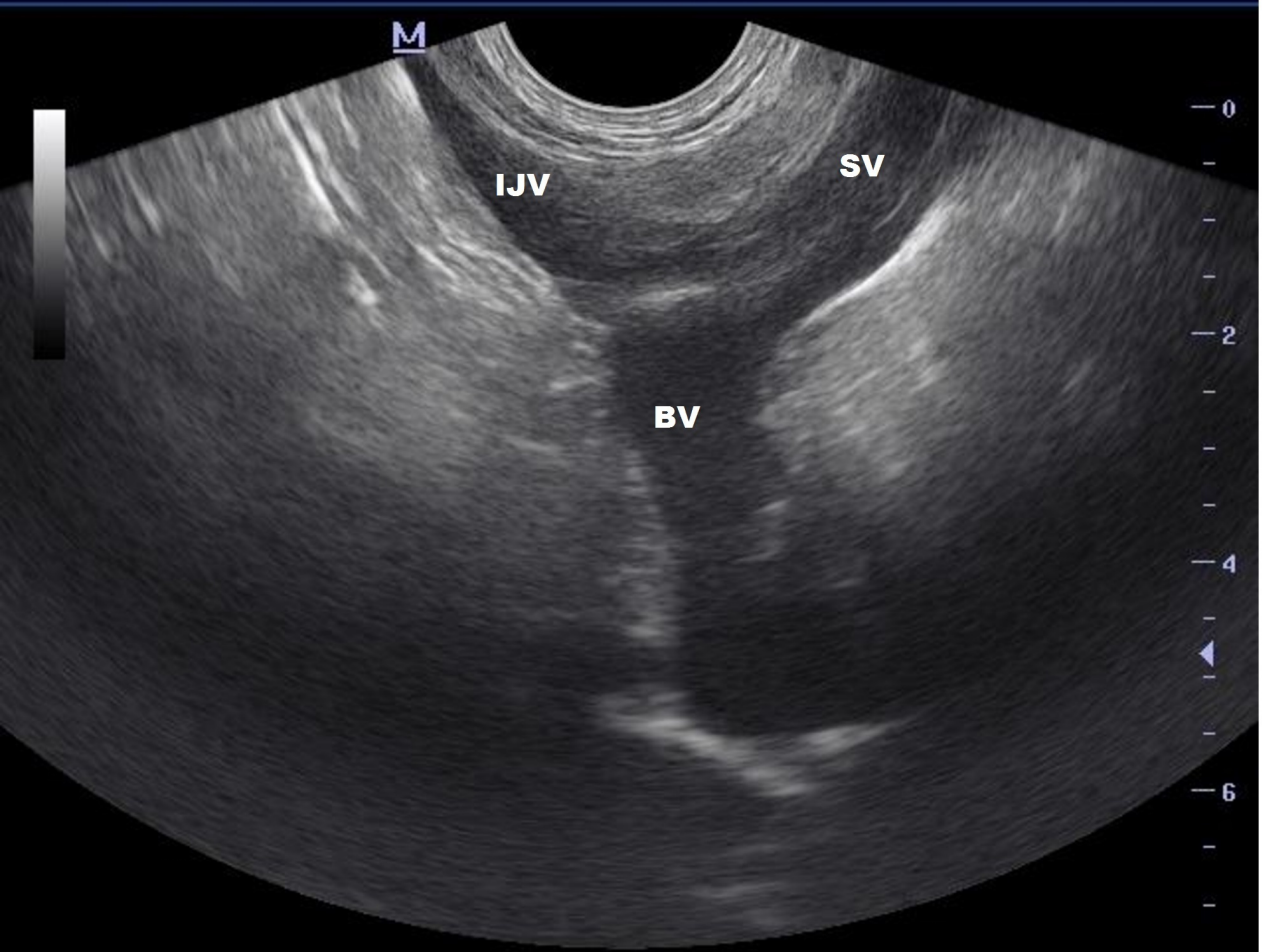
Y-Shape visualization of the three major veins IJV: internal jugular vein, BV: brachiocephalic vein, SV: subclavian vein

The omoclavicular acoustic window is described as the triangular fossa bordered by the clavicular head of the sternocleidomastoid muscle medially, the clavicle inferiorly, and the inferior belly of the omohyoid muscle posteriorly. We used a 5-8 MHz micro-convex endocavity probe (Mindray® Bio-Medical Electronics Co Ltd., Shenzhen, China) with an optimal imaging depth, which was set at 8 cm. With this method, a Y-shape is visualized, representing the jugular, subclavian, and brachiocephalic veins in the coronal plane (Figure 3).

**Figure 4:**
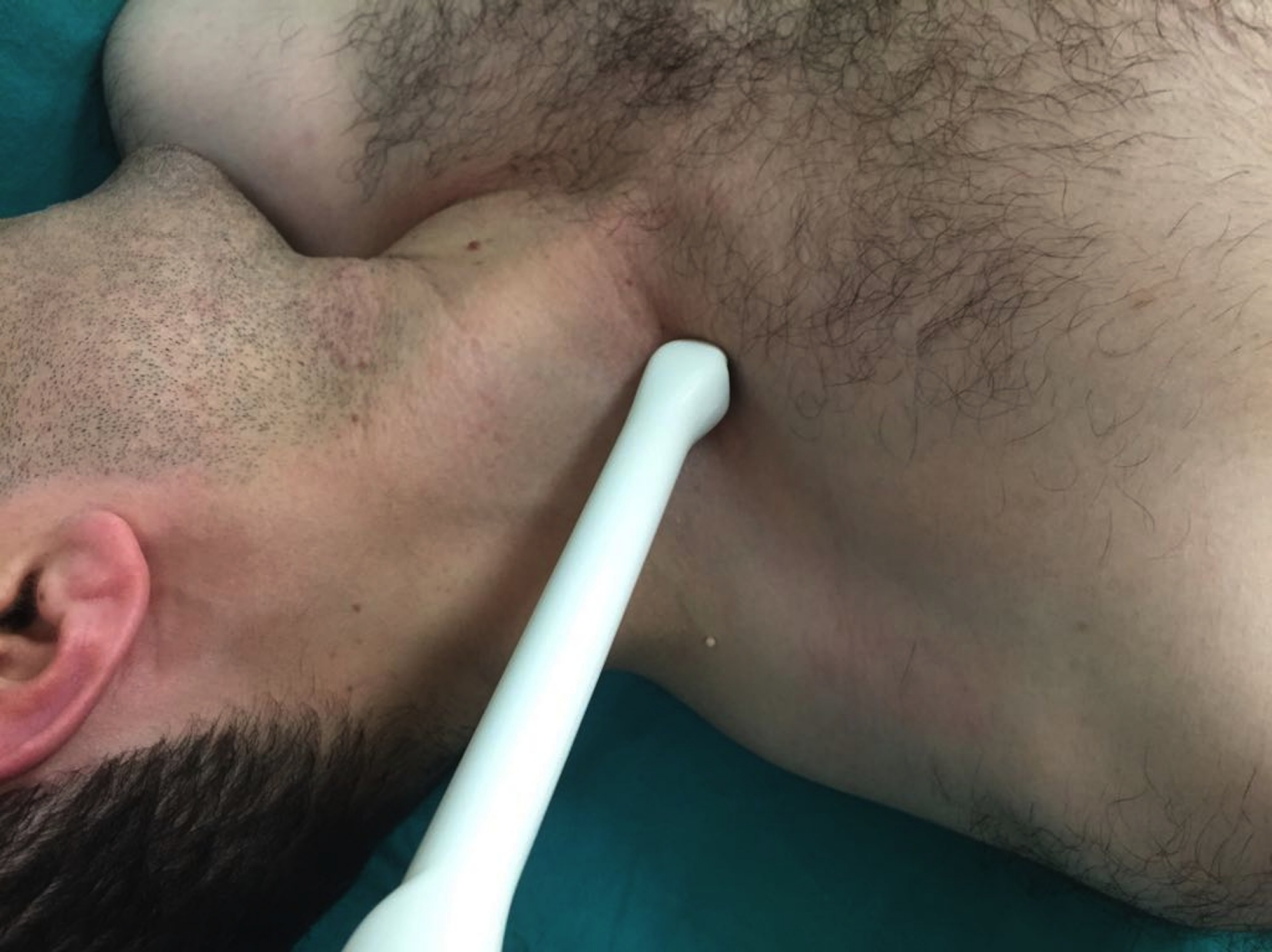
Photograph of the supraclavicular fossa in a volunteer Placement of the endocavity micro-convex US probe on the supraclavicular fossa for visualization of the jugular, subclavian, and brachiocephalic veins as a Y-shape

Another method to visualize the brachiocephalic vein with an endocavity micro-convex probe is placing it on the supraclavicular fossa in the coronal plane at about 30-45 degrees from the neck (Figure 4).

**Figure 5:**
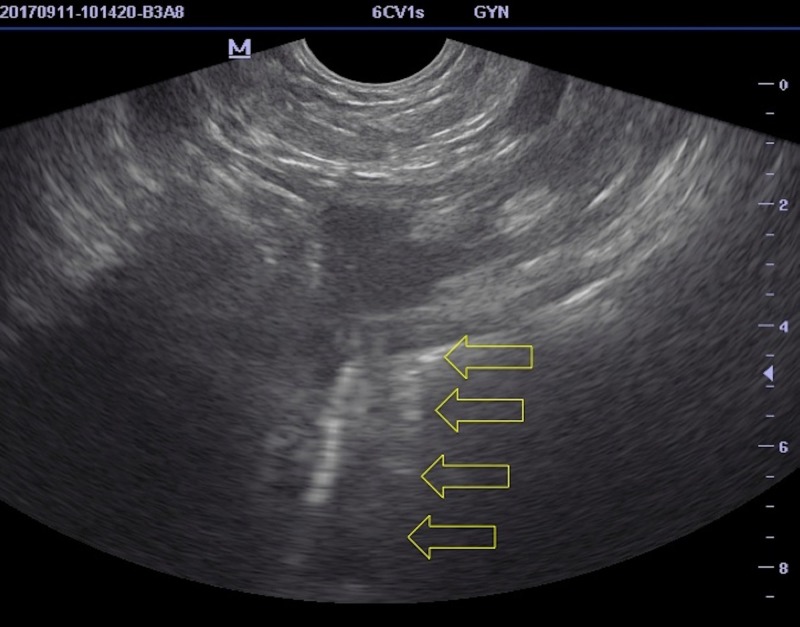
Image of the guidewire in the brachiocephalic vein obtained by the micro-convex endocavity probe in our case The arrows show the guidewire in the brachiocephalic vein.

The US probe can be slid anteriorly with the application of a slight alignment maneuver. The tilting maneuver could be applied as well to facilitate the visualization of the Y-shape of the veins. In our case, we successfully visualized the IJV, SV, and right BV forming a Y-shape (Figure 3) and confirmed that the guidewire was in the BV (Figure 5).

Then, we placed a triple-lumen 7 French central venous catheter (Arrow-Howes; Arrow International, Reading, PA, US) over the guidewire and fixed it in the proper position.

## Discussion

In this case, we successfully confirmed the placement of the guidewire in the brachiocephalic vein by using an endocavity micro-convex probe after a cannulation attempt to the jugular vein. The main advantage of the endocavitary micro-convex probe is the easy visualization of the brachiocephalic vein.

In general, linear probes have been used as the classical method in IJV catheterization [8]. Linear probes are often able to visualize the vasculature close to the skin surface due to their high resolution. However, Kim et al. noted a higher tissue penetration depth is possible when an endocavity micro-convex probe is used compared to a linear probe [9]. The advantage of using the endocavity micro-convex probe is that it can display structures deeper than 8 cm. Additionally, these probes also allow a wider field of view as Weber et al. reported [10]. Mallin et al. found the endocavitary micro-convex probe to be more advantageous because of its small footprint when placed on the supraclavicular fossa, which allows the performer a wider area for needle insertion and manipulations [11]. Using the supraclavicular fossa, a long axis image of the subclavian vein is obtained and its binding to the internal jugular vein and forming the brachiocephalic vein are observed. We preferred to visualize the BV through the omoclavicular acoustic window in our case, as we could obtain a clear image using this method.

Catheter malpositions and anatomic malformations can also be recognized by using an endocavity micro-convex probe that may not be visualized with a linear probe. Park et al. mentioned persistent left superior vena cava catheterization, which was diagnosed by chest radiography and could not be noticed by a linear US probe [12]. Habib et al. reported seven cases of catheter malpositions after ultrasound-guided catheterizations using a linear probe, which were also diagnosed by radiography [13]. The use of an endocavity micro-convex probe allows satisfactory images of the jugular, subclavian, and brachiocephalic veins so that the malpositions mentioned above may be diagnosed during the procedure.

## Conclusions

We confirmed the correct placement of the guidewire in the brachiocephalic vein using Y-shape visualization of the jugular, subclavian, and brachiocephalic veins with an endocavity micro-convex US probe. Visualization of the brachiocephalic vein up to a depth of 8 cm is possible using endocavity micro-convex probes. The verification could be performed during the catheterization procedure in the operating room without the necessitation of chest X-ray imaging.
